# Supplementary material for: H2AJ Is a Direct Androgen Receptor Target Gene That Regulates Androgen-Induced Cellular Senescence and Inhibits Mesenchymal Markers in Prostate Cancer Cells
Source: Cancers (Basel). 2025 Feb 25;17(5):791. doi: 10.3390/cancers17050791 (PMC11898987; doi:10.3390/cancers17050791)
Supplement: Supplementary file 1 [file cancers-17-00791-s001.zip › cancers-3490344-supplementary.pdf]

**Figure S1**

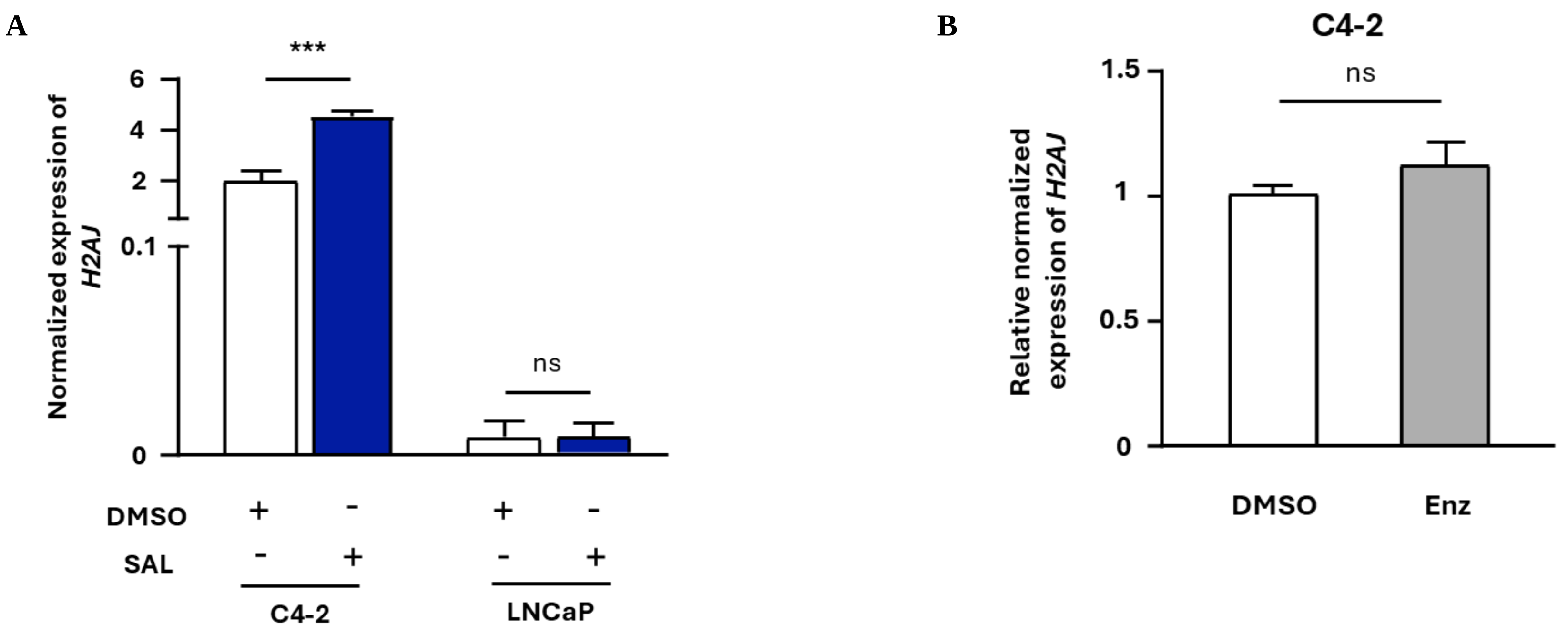


**Figure S1- Normalized mRNA level of *H2AJ* in different cell lines, upon AR agonist or antagonist, analyzed by qRT-PCR.**

**A**: Detection of *H2AJ* mRNA levels at DMSO and SAL treatment by qRT-PCR in C4-2 and LNCaP cell lines (n = 3). The mRNA levels of both housekeeping genes *α-Tubulin* and *TBP* were used for normalization of expression levels. **B**: Detection of *H2AJ* mRNA levels at DMSO and Enzalutamide (Enz) treatment by qRT-PCR in C4-2 cells (n = 2). The mRNA levels of both housekeeping genes *α-Tubulin* and *TBP* were used for normalization of expression levels. P value <0.001 = ***, ns= non-significant.

Figure S2

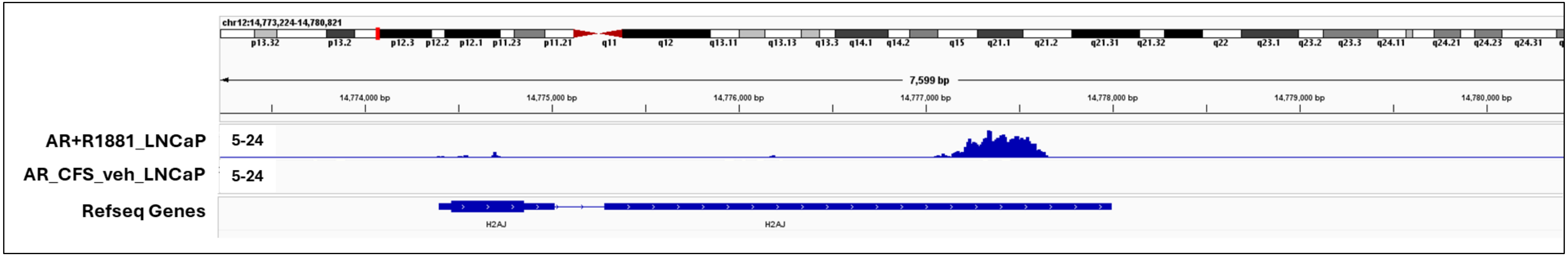


**Figure S2- Recruitment of AR to the *H2AJ* gene locus in LNCaP cells**

ChIP-seq data were analyzed for hormone-dependent recruitment of the AR to the *H2AJ* gene locus in LNCaP cells using IGV software for visualization. The synthetic androgen R1881 was used at supraphysiological levels. Veh, vehicle/solvent control (DMSO); CFS, charcoal-stripped serum. hg38 was used as a reference genome.

**Figure S3**

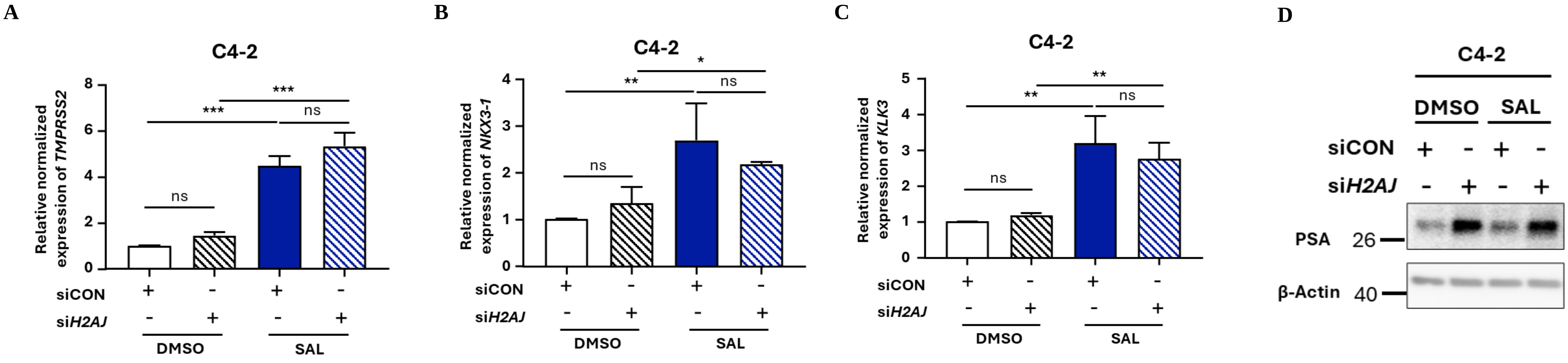


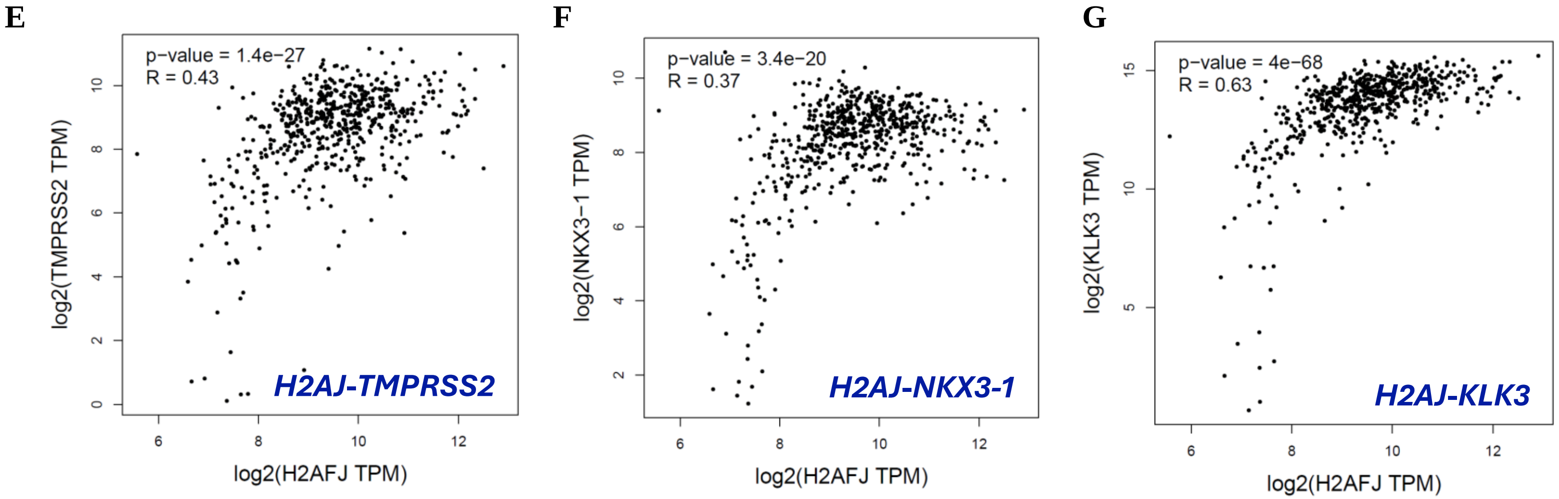


**Figure S3- Significant correlation analysis and regulation of AR direct target genes by H2AJ.**

**A**: qRT-PCR of RNA from cells with and without KD of *H2AJ* treated with DMSO or SAL to analyze *TMPRSS2* expression. **B**: The mRNA level of *NKX3-1*. **C**: The mRNA level of *KLK3* encoding the prostate specific antigen PSA. **D**: Western blot data analyzing protein level of PSA (29KDa) from si-control (siCON) and H2AJ knockdown (si*H2AJ*) samples with and without SAL treatment (n = 3). β-Actin served as loading control. **E**: *H2AJ-TMPRSS2* gene correlation based on PRAD and normal prostate samples datasets, **F**: *H2AJ-NKX3-1* gene correlation, **G**: *H2AJ-KLK3* gene correlation. Correlation analysis was performed using GEPIA with TCGA datasets. P value 0.001 = ***, <0.01 = **, <0.05 = *, ns= non-significant.

**Figure S4**

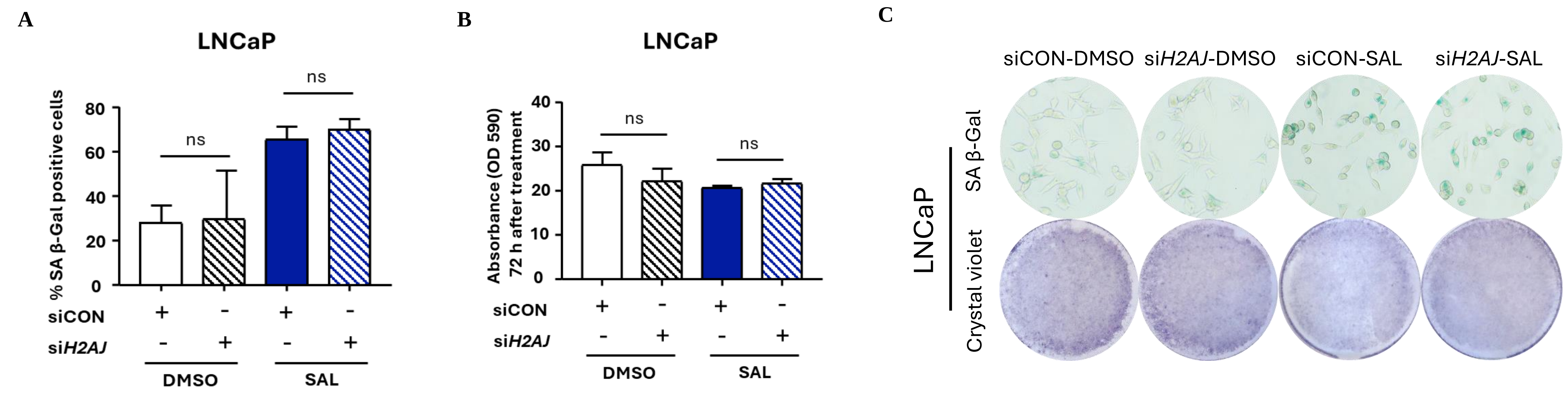


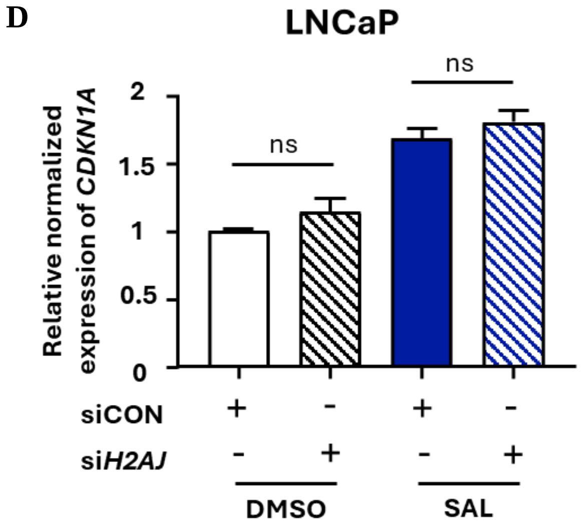


**Figure S4- *H2AJ* KD does not exhibit significant functionality in the LNCaP cell line.**

**A**: Quantification for SA β-Gal activity staining 3 days after treatment for siCON and *H2AJ* KD samples (n = 3). **B**: Quantification of crystal violet absorbance 3 days after treatment to analyze the growth of SAL-treated and H2AJ KD with and without SAL samples (n = 3). **C**: Representative pictures of SA β-Gal activity-stained cells and cell growth after 72 h of treatment. **D**: The expression level of *CDKN1A* mRNA in si-control (siCON) and *H2AJ* KD (si*H2AJ*) (n = 3). P value <0.001 = ***, <0.01 = **, <0.05 = *, ns= non-significant.

**Figure S5**

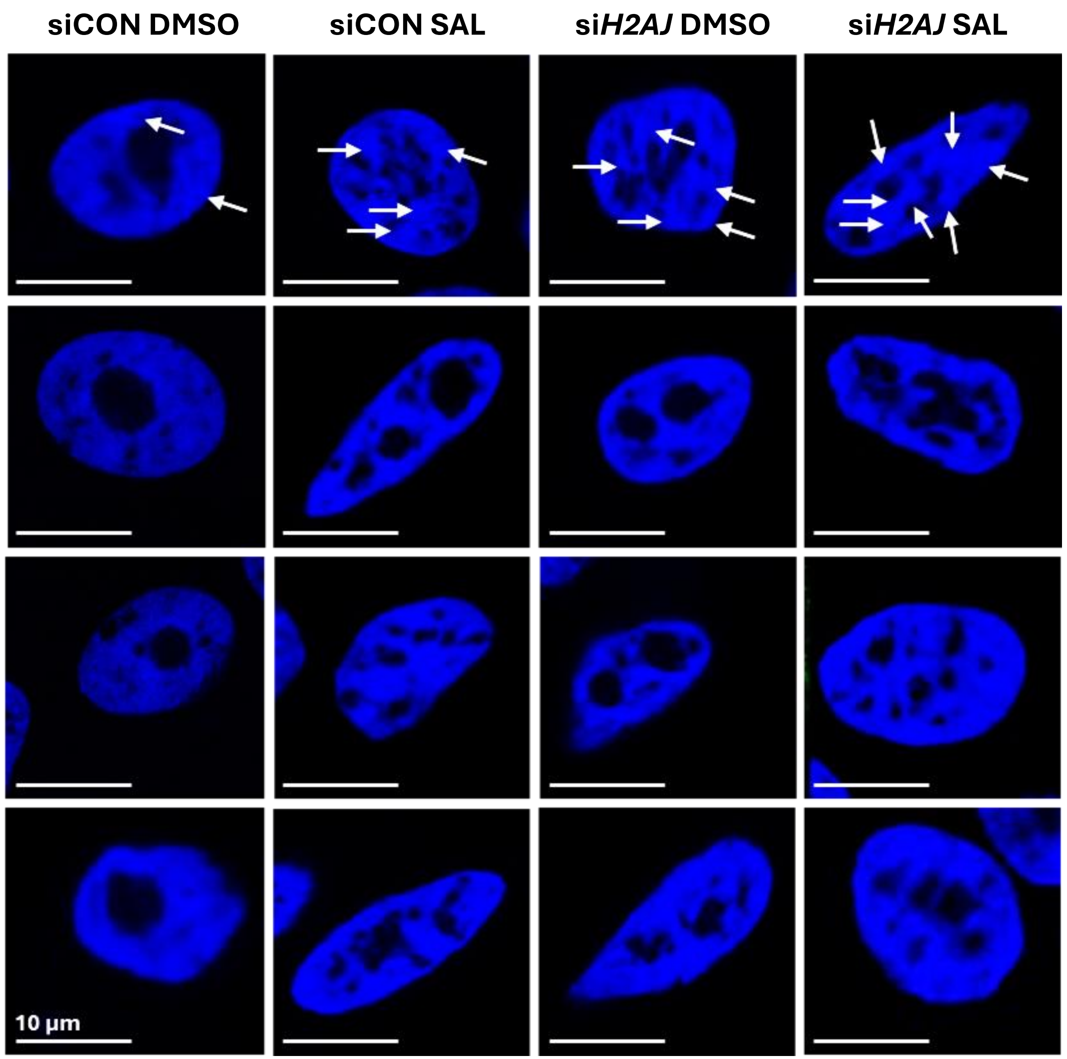


**Figure S5- H2AJ regulates SAHF formation.**

DAPI staining for SAHF (Senescence Associated heterochromatin Foci) in C4-2 cells of control cells (siCON) with and without SAL and for *H2AJ* KD samples with and without SAL treatment (n = 2). Four representative pictures are shown for each set. The white bars correspond to 10µm scaling.

**Figure S6**

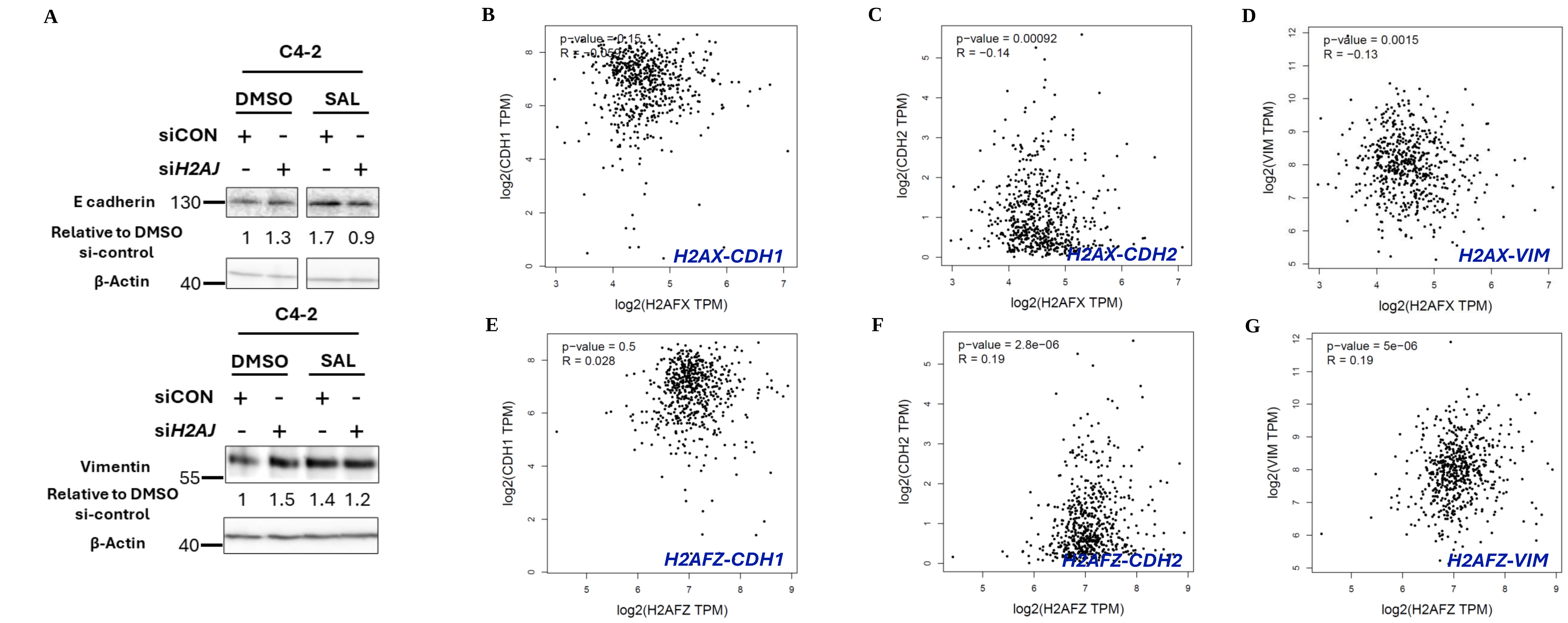


**Figure S6- Protein levels of EMT markers with and without *H2AJ* KD and correlation analysis between *H2AX* and *H2AFZ* with EMT markers in prostate adenocarcinomas.**

**A**: Protein levels of E-cadherin (130 KDa) and Vimentin (60 KDa) with and without *H2AJ* KD in C4-2 cells. **B-G**: Gene correlation based on prostate adenocarcinoma (PRAD) datasets for: **B**: *H2AX-CDH1* **C**: *H2AX-CDH2*. **D**: *H2AX-VIM*. **E**: *H2AFZ-CDH1* gene. **F**: *H2AFZ-CDH2*. **G**: *H2AFZ-VIM*.

**Figure S7**

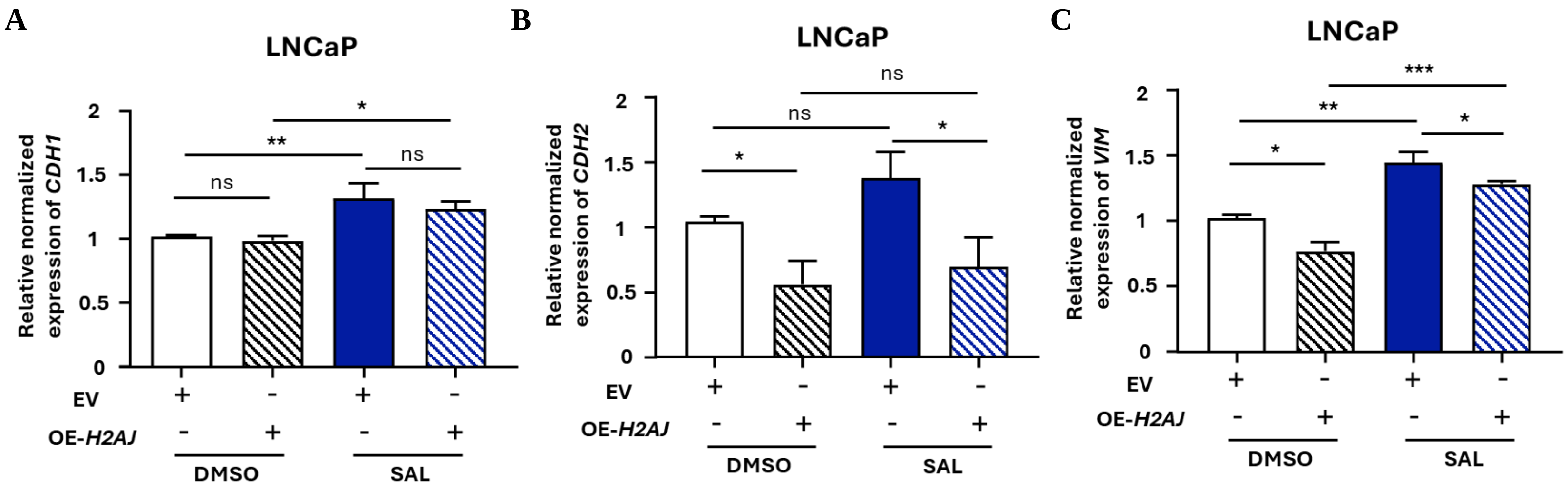


**Figure S7- *H2AJ* overexpression significantly reduces the expression levels of mesenchymal markers.**

**A**: qRT-PCR of *CDH1* mRNA encoding E-cadherin from LNCaP cells with or without *H2AJ* overexpression (OE-*H2AJ*) in combination with DMSO or SAL treatment. **B**: qRT-PCR for *CDH2* mRNA encoding N-cadherin with or without *H2AJ* overexpression in combination with DMSO or SAL treatment. **C**: qRT-PCR for *VIM* mRNA encoding Vimentin with or without *H2AJ* overexpression in combination with DMSO or SAL treatment. P value 0.001 = ***, <0.01 = **, <0.05 = *, ns= non-significant.

**Figure S8**

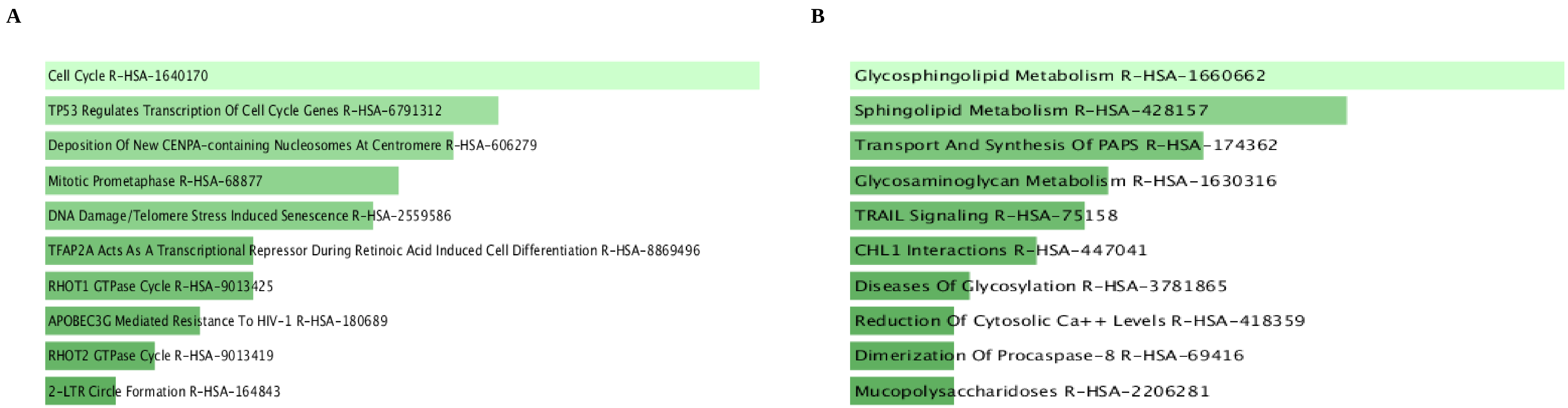


**Figure S8- Pathways identified for 64 differently and significantly regulated DEGs between the *H2AJ* KD samples and the cellular senescence score of PCa.**

**A**: List of significant pathways for 40 DEGs upregulated in *H2AJ* KD samples and downregulated in the senescence score. **B**: Identified list of significant pathways for 24 DEGs downregulated in *H2AJ* KD samples and upregulated in the senescence score. Color represents significance of pathways with the brighter the color, the more significant that term is. The length of the bar represents the significance of the indicated specific gene-set or term.
